# Nicotinamide riboside enhances liver regeneration via the MCART1/ASB3 axis in obesity-compromised rats

**DOI:** 10.1097/HC9.0000000000000866

**Published:** 2026-01-05

**Authors:** Hongbo Wang, Hai Li, Yang Liu, Xiangdong Wang, Chengjian He, Jian Huang, Yijun Zhang, Yefa Yang, Naijian Ge

**Affiliations:** Mini-Invasive Intervention Center, Third Affiliated Hospital of Naval Medical University/Shanghai Eastern Hepatobiliary Surgery Hospital, Shanghai, China

**Keywords:** ASB3, liver regeneration, MCART1, NAD^+^ metabolism, nicotinamide riboside, obesity, portal vein embolization

## Abstract

**Background:**

Obesity impairs liver regeneration by promoting chronic inflammation and metabolic dysfunction, especially in conditions like non-alcoholic fatty liver disease. Portal vein embolization (PVE), used to stimulate liver growth pre-hepatectomy, is less effective in obese subjects. Nicotinamide riboside (NR), a NAD^+^ precursor, improves mitochondrial function and lipid metabolism, but its role in liver regeneration under obese conditions remains unclear. Our study tried to investigate the effects and underlying mechanisms of NR on liver regeneration after PVE in high-fat diet (HFD)-induced obese rats.

**Methods:**

HFD-fed rats underwent PVE and were treated with or without NR. Liver regeneration was assessed by histology, 5-ethynyl-2′-deoxyuridine (EdU) incorporation, immunohistochemistry, and liver function tests. NAD^+^ levels were quantified to confirm NR activity. Proteomics, Kyoto Encyclopedia of Genes and Genomes (KEGG) pathway analysis, Gene Ontology (GO) analysis, quantitative real-time PCR (qPCR), and western blotting were used to explore molecular mechanisms, focusing on the MCART1/ASB3 axis.

**Results:**

Obesity impaired liver regeneration post-PVE, as evidenced by lipid accumulation, inflammation, reduced hepatocyte proliferation, and elevated liver enzymes. NR supplementation restored NAD^+^ levels, improved liver function, increased proliferative activity, and reduced steatosis. Mechanistically, NR upregulated MCART1 and ASB3 expression, promoting energy and lipid metabolism essential for regeneration.

**Conclusions:**

NR promotes liver regeneration after PVE in obese rats by enhancing NAD^+^-dependent metabolic pathways through the MCART1/ASB3 axis, offering a potential therapeutic strategy for obesity-associated liver dysfunction.

## INTRODUCTION

Obesity is defined as excess body fat, with body mass index (calculated as body weight in kilograms divided by height in square meters) being the most widely used clinical measure.^1^ It is now considered to be a serious global epidemic, affecting more than 2 billion people.^2^ Obesity elevates the morbidity of type 2 diabetes, hypertension, fatty liver disease, and obstructive sleep apnea.^3^ Particularly, the obesity epidemic is closely associated with a dramatic surge in the prevalence and severity of nonalcoholic fatty liver disease (NAFLD).^4^ NAFLD poses a significant threat to human health as it stands as the primary cause of chronic liver disease, which encompasses a wide spectrum of pathogenesis starting from excess fat in the liver (steatosis) that can progress to inflammation (steatohepatitis) and cirrhosis.^5^ Meanwhile, patients with NAFLD have a higher risk of cardiovascular mortality.^6^ This underscores the dire need for a granular understanding of the evolution of the disease and the factors related to both disease progression and regression.

Nicotinamide riboside (NR) is in wide use as a dietary supplement, which is a form of vitamin B3 and nicotinamide adenine dinucleotide (NAD^+^) precursor, and can be converted to bioavailable NAD^+^.^7^,^8^ NR has shown robust therapeutic benefits in ameliorating a variety of serious health conditions in humans, especially liver disease.^9^ In the liver of obese and diabetic KK mice, NR treatment improves glucose homeostasis, increases adiponectin, and lowers hepatic cholesterol; in addition, NR treatment can significantly downregulate the expression of hepatic pro-inflammatory markers, including tumor necrosis factor-alpha (TNF-α), interleukin (IL)-6, and IL-1 by modulating the NLRP3 inflammasome.^10^ Of much interest, the health benefits of NR are mediated through elevated NAD^+^ levels in the body.^11^ NAD^+^ is a crucial cofactor in redox metabolism and metabolic signaling, participating in redox reactions in central metabolic pathways, including glycolysis, oxidative phosphorylation, tricarboxylic acid cycle, and one-carbon metabolism, with its reduced form NADH.^12^,^13^,^14^ It is reported that hepatic NAD^+^ level is decreased in aged mice and humans with NAFLD; middle-age dominant-negative, enzymically inactive nicotinamide phosphoribosyltransferase transgenic mice given normal chow exhibit systemic NAD^+^ reduction and have moderate NAFLD-related phenotypes, such as enhanced oxidative stress, lipid accumulation, increased inflammation, and impaired insulin sensitivity in the liver; conversely, these NAFLD phenotypes driven by NAD^+^ deficiency or high-fat diet can be completely rescued by oral administration of NR.^15^ Furthermore, NR supplementation in mammalian cells and mouse tissues has been shown to increase NAD^+^ levels and activate SIRT1 and SIRT3, leading to enhanced oxidative metabolism and protecting against metabolic abnormalities due to a high-fat diet.^16^ Previous work has mainly focused on partial hepatectomy models, in which NR was reported to accelerate liver regeneration. However, partial hepatectomy does not fully recapitulate the clinical scenario of preoperative liver preparation in patients with NAFLD or obesity. In contrast, portal vein embolization (PVE) is a standard clinical procedure used to induce hypertrophy of the future liver remnant before major hepatectomy,^17^ and regeneration in non-embolized lobes occurs through redistribution of portal blood flow, increased shear stress, and upregulation of growth factors and cytokines such as HGF, VEGF, and IL-6.^18^ Therefore, the PVE model better reflects the regenerative challenge under metabolic stress than partial hepatectomy.

Importantly, the mechanisms linking NR to improved regeneration beyond NAD^+^ replenishment have not been fully clarified. In this study, we therefore established a PVE model in high-fat diet (HFD)-induced obese rats to investigate the role of NR in hepatic regeneration. Moreover, we sought to identify novel downstream mediators and report, for the first time, that the MCART1/ASB3 axis may contribute to NR-driven metabolic reprogramming and regenerative responses.

## METHODS

### Establishment of the HFD-induced obesity model in rats

Male Sprague–Dawley (SD) rats (22 weeks old) were purchased from SPF (Beijing) Biotechnology Co., Ltd. Obesity was induced by feeding the rats an HFD for 4 weeks. All animals were housed under controlled conditions with a temperature of 20–26 °C and a relative humidity of 40–70%. The HFD formulation consisted of 40% fat, 20% protein, and 30% carbohydrates.

### Animal grouping

The rats were randomly assigned to 5 groups, with 4 animals per group: control group (Control rats), obesity group (HFD-induced obesity rats), sham-operated control group (sham surgery without embolization), PVE group (PVE surgery performed in HFD-induced obese rats), and PVE+NR group (PVE surgery followed by NR intervention). Rats in the Sham, PVE, and PVE+NR groups were sacrificed on days 1, 3, 7, and 14 post-PVE. Blood and liver tissue samples were collected for subsequent analysis. To comprehensively evaluate the liver regeneration process, data were collected at these time points to observe the dynamic changes in hepatic regeneration.The care of animal and licensing guidelines under which the study was performed and report these in accordance with the ARRIVE.

### Oil red O staining

Liver lobes were embedded in optimal cutting temperature (OCT) compound and sectioned using a cryostat (CM1950, Leica). Frozen sections were fixed in 4% paraformaldehyde for 10 minutes and washed with double-distilled water. The sections were then rinsed with 60% isopropanol for 2 minutes, followed by staining with Oil Red O solution (G1015-100 ML, Servicebio) for 10 minutes. Excess stain was removed with 60% isopropanol, and the sections were washed with double-distilled water. Hematoxylin staining was performed for 3 minutes, followed by differentiation in ethanol-hydrochloric acid solution for 10 seconds. The slides were rinsed briefly, counterstained in tap water for 3 minutes, and washed under running water. Finally, the sections were mounted with glycerol gelatin and imaged under a microscope.

### Hematoxylin and eosin staining

Paraffin-embedded liver sections were deparaffinized in xylene and rehydrated through a graded ethanol series. The sections were then stained with hematoxylin, followed by counterstaining with eosin. After dehydration, the slides were mounted with coverslips using neutral resin and observed under a microscope.

### 5-Ethynyl-2′-deoxyuridine staining

5-Ethynyl-2′-deoxyuridine (EdU) was administered via intraperitoneal injection 24 hours before euthanasia. Liver tissues were dehydrated in graded ethanol solutions (75%, 85%, 95% I, and 100% I) for 60 minutes each, followed by clearance in xylene I and II for 45 minutes each. The tissues were then embedded in paraffin and sectioned. The EdU fluorescence signal was examined under a fluorescence microscope to evaluate the proliferative activity of hepatocytes in liver lobes.

### Immunohistochemistry

Paraffin-embedded liver tissue sections were deparaffinized, rehydrated, and subjected to antigen retrieval, endogenous peroxidase inactivation, and nonspecific blocking. Primary antibodies were then applied to each slide: Ki67 (ab16667, Abcam, 1:200), ASB3 (orb1150858, Biorbyt, 1:150), rabbit anti-Caspase-3 (1:100), and rabbit anti-Caspase-9 (1:100), followed by overnight incubation at 4 °C. After washing with PBS, the sections were incubated with horseradish peroxidase (HRP)-conjugated goat anti-rabbit IgG (H+L) (1:100) for 30 minutes. Hematoxylin counterstaining was performed for 3 minutes, followed by dehydration using a graded ethanol series (80%, 95%, absolute ethanol I, absolute ethanol II), and clearing with xylene I and II. The sections were mounted with neutral resin and observed under a microscope. DAB was used for chromogenic detection, with nuclei appearing blue due to hematoxylin staining, while DAB-positive signals were visualized as brown staining. The number of Ki67-positive cells was qualitatively assessed by visual inspection, and staining intensity as well as positive cell distribution patterns were compared among different experimental groups.

### Liver function assays

Serum samples were obtained by centrifugation of collected rat blood. Liver function parameters, including total bilirubin (TBIL, 70182), direct bilirubin (DBIL, 70184), albumin (ALB, 70113), aspartate aminotransferase (AST, 70110), and alanine aminotransferase (ALT, 70111), were measured using a fully automated biochemical analyzer (BK-600, Shandong Biok).

### PVE model establishment

PVE modeling was performed under anesthesia. A midline incision was made below the xiphoid process, and the hepatic ligaments were carefully dissected to expose the portal vein. The left branch of the portal vein was isolated, and its distal end was occluded. A 0.2 mL embolizing agent [a 1:3 mixture of N-butyl cyanoacrylate (NBCA) and iodized oil] was injected into the left portal vein branch. The abdominal incision was then closed. Rats in the sham group underwent the same surgical procedures, except that no embolizing agent was injected. Rats in the PVE+NR group received drinking water supplemented with 3 mg/mL NR immediately after surgery. The NR solution was replaced every 3 days. The remaining groups had access to normal drinking water.

### NAD biochemical assay

Liver tissue samples were lysed, and NAD^+^/NADH levels were quantified using an NAD(H) assay kit (A114-1-1, Nanjing Jiancheng) according to the manufacturer’s instructions. Measurements were performed using a fully automated microplate reader (WD-2012B, Beijing Liuyi).

### Liver-to-body weight ratio calculation

The liver-to-body weight ratio (%) was calculated as follows: Liver-to-body weight ratio=(Body weight/Liver weight)×100%.

### Proteomics analysis

Protein expression levels were statistically analyzed using fold change analysis and t-tests to identify differentially expressed proteins.

### Kyoto Encyclopedia of Genes and Genomes enrichment analysis

Differentially expressed proteins were subjected to Kyoto Encyclopedia of Genes and Genomes (KEGG) enrichment analysis. The identified proteins were submitted to the KEGG database for pathway analysis. Using the species protein dataset as the background list and the differentially expressed proteins as the candidate list, hypergeometric distribution testing was employed to calculate the significance (*p*-value) of functional enrichment within the differentially expressed protein set. The *p*-values were then adjusted for false discovery rate (FDR) correction to obtain the final enrichment results.

### Gene Ontology enrichment analysis

GO enrichment analysis was performed on the differentially expressed proteins by submitting them to the Gene Ontology (GO) database for functional annotation. The species protein dataset was used as the background list, while the differentially expressed proteins served as the candidate list extracted from the background. Hypergeometric distribution testing was conducted to assess the statistical significance of enrichment (*p*-value) within the functional sets. FDR correction was applied to the *p*-values to ensure statistical robustness.

### Western blotting

Liver tissue samples were lysed and homogenized for protein extraction. Total protein concentrations were quantified using a bicinchoninic acid (BCA) protein assay kit. After denaturation, proteins were subjected to sodium dodecyl sulfate-polyacrylamide gel electrophoresis (SDS-PAGE) and transferred onto a polyvinylidene difluoride (PVDF) membrane (Millipore) under a constant current of 300 mA. The membrane was blocked with non-fat milk, followed by overnight incubation with primary antibodies at 4 °C. The next day, the membrane was incubated with secondary antibodies at room temperature. The PVDF membrane was then soaked in enhanced chemiluminescence (ECL) reagent and imaged using an ultra-sensitive chemiluminescence imaging system. The antibodies and their corresponding dilution ratios are listed in Table 1.

**TABLE 1:** Antibodies used for WB

Antibodies	Dilution
Mouse anti-β-actin (HC201, TransGen Biotech)	1:2000
Rabbit anti-MCART1 (bs-24079R, Bioss)	1:1000
Rabbit anti-ASB3 (BIORBYT, orb459213)	1:200

Abbreviation: WB, western blotting.

### Quantitative real-time PCR

Total RNA was extracted from liver tissue using the Trizon reagent (CW0580S, CWBIO), followed by mRNA purification using the RNA SuperPure Extraction Kit (CW0581M, CWBIO). The concentration and purity of mRNA were determined using a UV-visible spectrophotometer (NP80, NanoPhotometer) by measuring the OD260/OD280 ratio. Complementary DNA (cDNA) was synthesized from the extracted RNA using a reverse transcription kit (R223-01, Vazyme). Quantitative real-time PCR (qRT-PCR) was performed using a real-time fluorescence PCR system (CFXConnect, Bio-Rad, Shanghai, China). The PCR reaction conditions were as follows: pre-denaturation at 95 °C for 10 minutes, and a total of cycles of denaturation at 95 °C for 10 seconds, annealing at 58 °C for 30 seconds, extension at 72 °C for 30 seconds. β-Actin was used as the internal reference gene, and the relative gene expression levels were calculated using the 2^−ΔΔCt^ method. The primer sequences used for qRT-PCR are listed in Table 2.

**TABLE 2:** PCR primer sequences

	Primer sequences (5’–3’)
GAPDH F	GACAACTTTGGCATCGTGGA
GAPDH R	ATGCAGGGATGATGTTCTGG
ASB3 F	TGCTTGCTGGATTTGACCC
ASB3 R	TGCTGAAGAGCGAAAGAGA

### Statistical analysis

Statistical analysis and data visualization were conducted using GraphPad Prism 9.0.0. All experiments were performed in triplicate, and quantitative data were expressed as mean ± standard deviation (X̄ ± SD). One-way analysis of variance (ANOVA) was used to compare differences among multiple groups, with a significance threshold of α=0.05. A *p*-value <0.05 was considered statistically significant.

## RESULTS

### Liver inflammation is exacerbated, and liver regeneration is delayed in HFD-induced obese rats compared with normal rats

**FIGURE 1:**
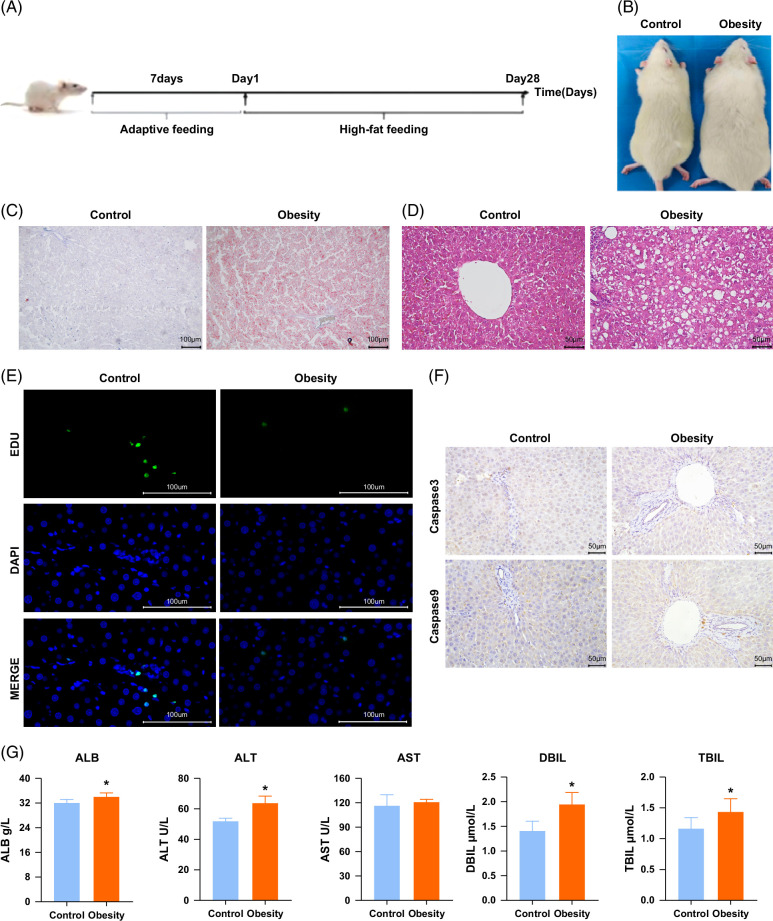
Exacerbated liver inflammation and impaired liver regeneration in high-fat diet-induced obese rats compared with normal rats. (A) Schematic representation of the modeling process; (B) comparison of obese and normal rats; (C) Oil Red O staining; (D) hematoxylin–eosin (H&E) staining; (E) EdU staining; (F) immunohistochemistry (IHC) analysis of caspase-3 and caspase-9 expression; (G) liver function assays. *p*<0.05. Abbreviations: ALB, albumin; ALT, alanine aminotransferase; AST, aspartate aminotransferase; DBIL, direct bilirubin; EdU, 5-ethynyl-2′-deoxyuridine (proliferation marker); TBIL, total bilirubin.

An HFD-induced obese rat model was successfully established following the modeling protocol illustrated in Figure 1A. Compared with the control group, rats in the obesity group exhibited a significant increase in body weight (Figure 1B), confirming the successful induction of obesity. Histological analyses further validated obesity-associated hepatic alterations. Oil Red O staining and hematoxylin and eosin (H&E) staining demonstrated excessive lipid droplet accumulation and increased inflammatory infiltration in the liver of rats in the obesity group compared with the control group (Figures 1C, D). EdU staining results revealed a marked reduction in the number of hepatocytes entering the proliferative phase in rats in the obesity group compared with the control group (Figure 1E). Immunohistochemistry (IHC) analysis further indicated that caspase-3 and caspase-9 expression levels were significantly downregulated in the obesity group, suggesting impaired liver regeneration in obese rats (Figure 1F). Serum liver function assays showed that key liver function indicators were significantly elevated in rats in the obesity group compared with the control group, exceeding the normal physiological range (Figure 1G). Collectively, these results indicate that HFD-induced obesity leads to aggravated liver inflammation and delayed liver regeneration in rats compared with normal controls.

### NR promotes liver function recovery following PVE in HFD-induced obese rats

**FIGURE 2:**
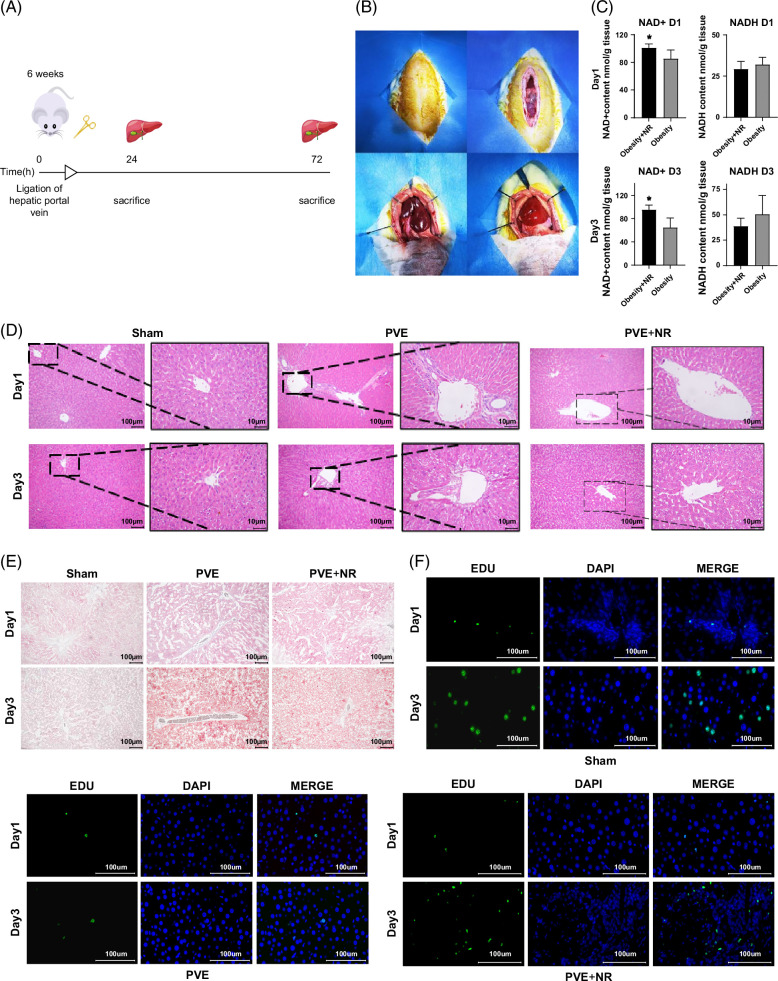
Nicotinamide riboside promotes liver function recovery following PVE in high-fat diet-induced obese rats. (A) Schematic representation of the modeling process; (B) construction of the PVE rat model; (C) NAD biochemical detection; (D) Oil Red O staining; (E) hematoxylin–eosin (H&E) staining; (F) EdU staining. *p*<0.05. Abbreviations: EdU, 5-ethynyl-2′-deoxyuridine (proliferation marker); NAD^+^, nicotinamide adenine dinucleotide; NR, nicotinamide riboside; PVE, portal vein embolization.

Based on the established HFD-induced obese rat model, PVE surgery was performed (Figure 2A). Following embolization, the left lateral and left medial lobes of the liver turned dark red, while the non-embolized lobes, including the right anterior, papillary, and caudate lobes, remained bright red, confirming the successful establishment of the PVE model (Figure 2B). NR supplementation was administered to PVE model rats, and biochemical analysis of NAD levels indicated a significant increase in hepatic NAD levels following NR supplementation, while the reduced form, NADH, correspondingly decreased. These results confirmed the successful establishment of the PVE+NR obese rat model (Figure 2C). Histological analysis using Oil Red O and H&E staining revealed prominent inflammatory infiltration and lipid droplet accumulation in the PVE group compared with the sham group. However, in the PVE+NR group, lipid droplet accumulation was reduced, and inflammatory infiltration was notably alleviated compared with the PVE group (Figures 2D, E). EdU staining demonstrated that stem cells in the PVE+NR group exhibited enhanced proliferative activity compared with both the sham and PVE groups. Notably, hepatocyte proliferation peaked on postoperative day 3, followed by a gradual decline. Collectively, these results indicate that NR facilitates liver function recovery following PVE in HFD-induced obese rats.

### NR enhances liver regeneration after PVE in HFD-induced obese rats

**FIGURE 3:**
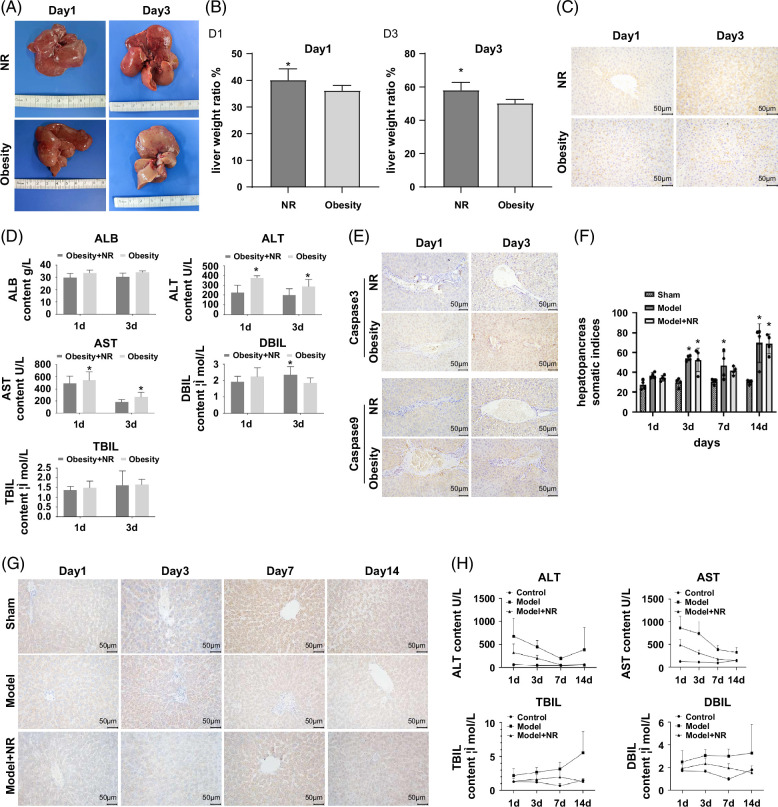
Nicotinamide riboside enhances liver regeneration following PVE in high-fat diet-induced obese rats. (A) Gross anatomical evaluation of rat liver; (B/F) liver-to-body weight ratio; (C/G) immunohistochemical Ki67 staining; (D/H) liver function assays; (E) immunohistochemistry analysis of caspase-3 and caspase-9 expression. *p*<0.05. Abbreviations: ALB, albumin; ALT, alanine aminotransferase; AST, aspartate aminotransferase; DBIL, direct bilirubin; NR, nicotinamide riboside; TBIL, total bilirubin.

Macroscopic and histological assessments of liver regeneration revealed that in the Control group, the liver surface appeared pale and rough, with irregularly distributed red spots. In contrast, rats supplemented with NR exhibited a more uniform and reddish liver surface, along with significant hepatic proliferation (Figure 3A). The liver-to-body weight ratio is a key parameter reflecting the extent of liver regeneration. During postoperative recovery, the liver-to-body weight ratio in the NR group was significantly higher than that in the control group on both postoperative day 1 and day 3, indicating an accelerated liver regeneration rate following NR supplementation (Figure 3B). This finding was further supported by Ki67 staining, which demonstrated enhanced hepatocyte proliferation in the NR group (Figure 3C). Serum liver function tests also indicated that NR supplementation improved liver function, as evidenced by liver function parameters returning closer to normal levels compared with the control group (Figure 3D). In addition, IHC analysis showed significantly increased caspase-3 and caspase-9 expression in the NR group, further confirming an accelerated liver regeneration process (Figure 3E). To further investigate the regulatory effect of NR on liver regeneration over an extended time frame, multiple time points were selected (days 1, 3, 7, and 14 post-PVE) to evaluate the progression of liver regeneration. Compared with the sham group, the PVE and PVE+NR groups exhibited a significant increase in liver-to-body weight ratio at all observed time points, indicating active liver regeneration (Figure 3F). Consistently, Ki67 staining confirmed that both the PVE and PVE+NR groups exhibited high proliferative activity at all time points, with pronounced liver regeneration (Figure 3G). Furthermore, serum ALT and AST levels in the PVE group remained significantly elevated on postoperative days 1, 3, 7, and 14 compared with the sham group. However, in the PVE+NR group, ALT and AST levels were significantly lower than those in the PVE group at all time points, displaying an overall decreasing trend (Figure 3H). Taken together, these findings suggest that NR effectively promotes liver regeneration following PVE in HFD-induced obese rats.

### NR may regulate liver regeneration via lipid metabolism pathways

To further elucidate the downstream molecular mechanisms by which NR regulates liver regeneration, a proteomic analysis was performed on the regenerating liver lobes of NR-treated and PVE-only rats on postoperative day 3. The normalized data exhibited an approximately normal distribution (Figure 4A). A volcano plot was generated based on fold change analysis and *t* test statistics, revealing significant differences in protein expression between the NR and PVE groups in the non-embolized liver lobes. A total of 144 differentially expressed proteins were identified, including 76 upregulated and 68 downregulated proteins (Figure 4B). Principal component analysis (PCA) demonstrated a clear separation trend between the NR and PVE groups, suggesting distinct metabolic pathway alterations between the 2 groups (Figure 4C). A hierarchical clustering heatmap of significantly differentially expressed proteins suggested potential involvement of lipid metabolism–related regulators, including pathways linked to STEAP4, which may influence lipid accumulation and inflammatory responses during regeneration (Figure 4D). KEGG and GO enrichment analyses further indicated that the effects of NR may involve lipid metabolism, nucleotide metabolism, small molecule metabolism, and organic acid metabolism. Notably, organic acid metabolism was significantly enriched in GO analysis, implying a possible association between NR treatment and fatty acid metabolism (Figures 4E, F). Although these findings provide important clues, they remain exploratory and require further validation by targeted molecular assays to confirm the specific pathways involved.

FIGURE 4Nicotinamide riboside may regulate liver regeneration via lipid metabolism pathways. (A) Proteomic analysis; (B) volcano plot; (C, D) PCA and protein normalization; (E) KEGG analysis; (F) GO analysis. Abbreviations: KEGG, Kyoto Encyclopedia of Genes and Genomes; GO, Gene Ontology; PCA, principal component analysis.

**Figure:**
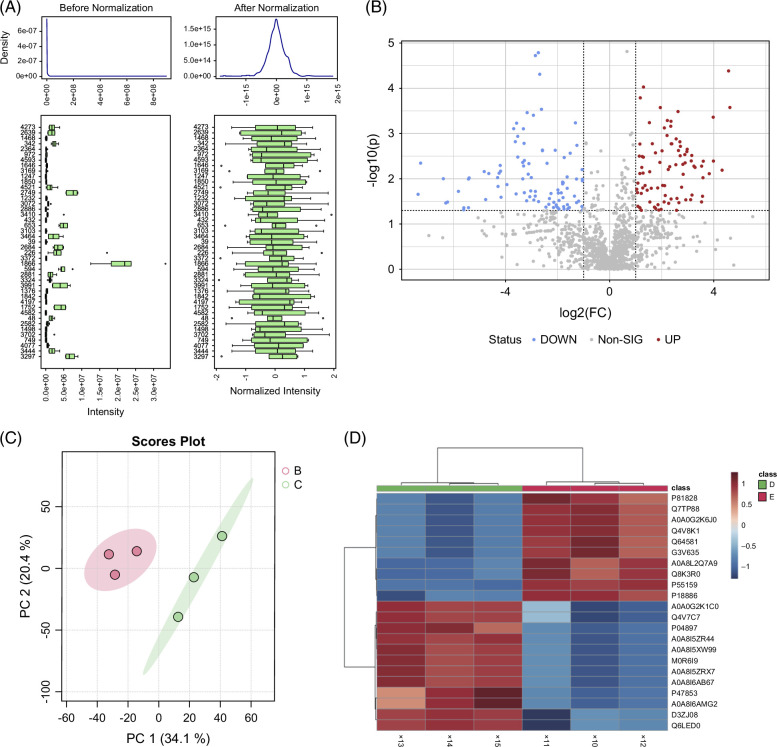

**Figure:**
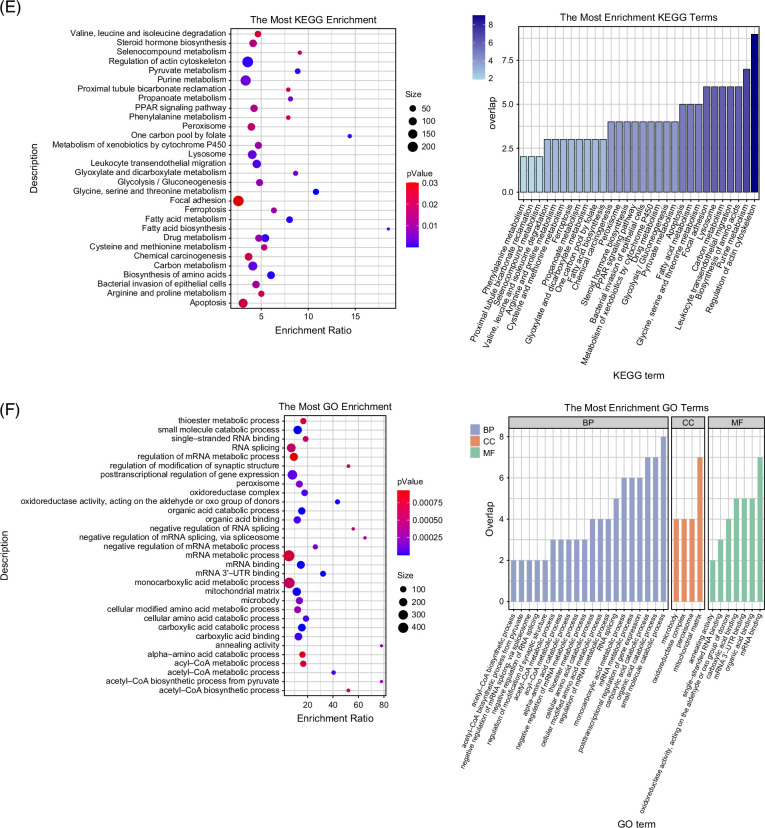

### NR modulates MCART1/ASB3 to influence metabolism and promote liver regeneration after PVE in HFD-induced obese rats

**FIGURE 5:**
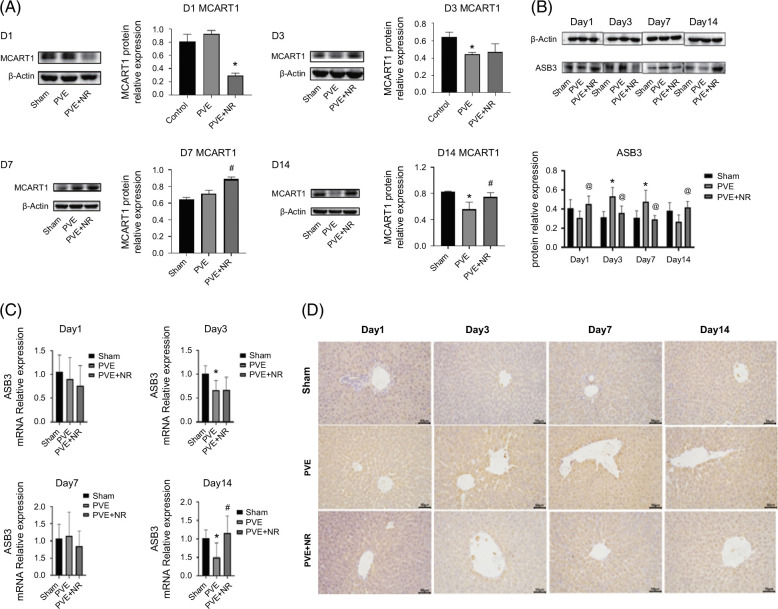
Nicotinamide riboside modulates MCART1/ASB3 to influence metabolism and promote liver regeneration after PVE in high-fat diet-induced obese rats. (A, B) Western blotting analysis of MCART1 and ASB3 protein expression; (C) PCR analysis of ASB3 expression; (D) immunohistochemistry analysis of ASB3 expression. *p*<0.05. Abbreviations: NR, nicotinamide riboside; PVE, portal vein embolization.

After identifying that NR may influence liver regeneration through lipid metabolism pathways, we further examined the expression of MCART1, a mitochondrial NAD/energy metabolism-associated protein, and ASB3, a lipid metabolism–related protein. Western blotting (WB) analysis revealed that compared with the sham group, MCART1 expression decreased in the PVE group on postoperative day 14, whereas in the PVE+NR group, MCART1 expression appeared relatively higher at the same time point, suggesting a possible role of NR in restoring mitochondrial energy metabolism (Figure 5A). For ASB3 expression, WB analysis demonstrated that compared with the sham group, ASB3 levels in the PVE group increased significantly on postoperative days 3 and 7, followed by a decline on day 14. However, in the PVE+NR group, ASB3 expression showed a different trend: it was significantly increased on day 1, followed by a notable decrease on days 3 and 7, and then a substantial increase on day 14 (Figure 5B).

These findings indicate that NR treatment is associated with altered expression patterns of MCART1 and ASB3 during regeneration after PVE. While this correlation suggests potential involvement of these proteins in NR-mediated metabolic regulation, we acknowledge that the evidence remains associative and does not establish a direct causal link. Further studies will be required to clarify whether modulation of MCART1 and ASB3 is mechanistically responsible for the observed regenerative effects.

## DISCUSSION

This study demonstrated, through an in vivo model, that NR could potentially repress hepatocyte apoptosis and improve energy and fat metabolism in the liver, noticeably promoting liver repair and regeneration after injury. An increase in NAD^+^ level was the underlying cause of this therapeutic benefit of NR. Therefore, this work provides strong experimental evidence for investigating the clinical application of NR for the treatment or prevention of metabolic liver disease. Unlike previous studies focusing on partial hepatectomy models, our work employed a portal vein embolization PVE model under obesity/NAFLD conditions, which better mimics the clinical setting where regeneration is often impaired.

Decreased hepatocyte viability and increased cell apoptosis evidence the emergence of hepatocyte injury; strikingly, the supplementation of NR can improve the high-glucose-induced hepatocyte injury by enhancing cell viability and suppressing cell apoptosis.^19^,^20^ Consistent with this finding, our current results suggest that NR could significantly potentiate hepatocyte proliferation and impair cell apoptosis in a rat model of high-fat diet-induced NAFLD and PVE. This extends prior findings by showing that NR enhances hepatocyte proliferation specifically in regenerating lobes after PVE, suggesting translational relevance for obese patients undergoing pre-hepatectomy PVE.

Moreover, it is well accepted that oxidative stress and inflammatory response represent the major risk factors involved in the pathogenesis of NAFLD; their inhibition may thus provide protection against NAFLD.^21^,^22^ Specifically, in the context of NAFLD, the lipid accumulation in hepatocytes (hepatic macro-vesicular steatosis), driven by increased fat intake, occurs and increases the vulnerability of the liver to a variety of secondary insults such as oxidative stress, and the subsequent activation of inflammatory pathways by the production of pro-inflammatory cytokines, including IL-1, IL-6, and TNF-α through toll-like receptor 4 activation, by Kupffer cells or adipokines from adipocytes, dysregulated hepatocyte apoptosis and hepatic stellate cell activation. The consequent fibrosis progresses from portal/periportal fibrosis to bridging fibrosis, and then cirrhotic remodeling with liver failure and, eventually, hepatocarcinogenesis.^23^,^24^,^25^ In line with these mechanisms, our results showed that NR supplementation alleviated lipid accumulation and reduced inflammatory infiltration in regenerating livers following PVE.

In addition to increased oxidative stress and inflammatory response, liver injury is also intimately associated with impaired fat and energy metabolism, which induces insufficient energy supply and therefore an impaired capacity of the liver to regenerate.^26^,^27^ The liver plays a central role in metabolic homeostasis, including glucose and lipid homeostasis, and amino acid metabolism; disrupted hepatic metabolic homeostasis is closely involved in the mechanism underlying the development of NAFLD.^28^This is further explained by the fact that an imbalance between energy intake and expenditure leads to insulin resistance in various tissues and alteration of the gut microbiota, contributing to fat accumulation in the liver. This fat accumulation process is frequently accompanied by intracellular damage and hepatic insulin resistance, which further exacerbate inflammation, fibrosis, and carcinogenesis.^29^,^30^ In this case, promotion of hepatic fat and energy metabolism may be beneficial for the prevention and alleviation of NAFLD.^31^

NR improves liver health in a broad array of contexts through regulation of the above-revealed causative factors; for instance, it delays NAFLD progression by potently reducing fat accumulation through a mechanism that involves induction of the mitochondrial unfolded protein response in a mouse model.^32^ Moreover, it decreases inflammation at least in part by downregulating the activity of the NLRP3 inflammasome, and consequently lessens the development of liver fibrosis.^33^,^34^ Much in line with current results, there is increasing evidence supporting that the health benefits of NR are mainly attributable to the enhancement of NAD^+^ biosynthesis.^19^ In the regenerating liver following partial hepatectomy, NR reduces lipid accumulation, increases hepatic adenosine triphosphate content, facilitates hepatocyte replication, and triggers faster liver weight regain by increasing hepatic NAD^+^ level.^34^ Our data build upon these observations by demonstrating that NR also accelerates regeneration after PVE in obesity-induced NAFLD rats, which has greater clinical relevance than partial hepatectomy models. Importantly, NAD^+^ bioavailability has been found to be impaired in the event of metabolic disease, including diet-induced obesity, NAFLD, and hepatocellular carcinoma.^35^,^36^,^37^ Interestingly, both DNA damage and tumorigenesis can be arrested when NR is provided in the diet to restore NAD^+^ pools in a mouse model of hepatocellular carcinoma.^38^ These positive effects of NAD^+^ level are primarily due to its crucial role in maintaining energy homeostasis by functioning as a substrate for different enzyme families, such as sirtuins and poly(ADP-ribose) polymerases.^35^

Of particular novelty, we identified the MCART1/ASB3 axis as a potential downstream mediator of NR action. NR supplementation restored MCART1 expression, which is essential for mitochondrial NAD transport and energy metabolism, and modulated the dynamic expression of ASB3, a lipid metabolism–related protein. Although functional validation is still required, this is the first report implicating MCART1 and ASB3 in NR-driven liver regeneration, thereby expanding mechanistic understanding beyond the classical NAD^+^–sirtuin pathway.

In summary, this study provides evidence for the role of NR in the metabolic function of the liver and protection against liver injury. Importantly, we demonstrated that NR supplementation not only improved metabolic dysfunction but also enhanced regeneration after PVE in obesity-compromised rats, a clinically relevant model. We further identified MCART1 and ASB3 as novel downstream effectors, providing new mechanistic insights. Altogether, our findings highlight the translational potential of NR as an adjuvant to improve liver regeneration in obese patients undergoing pre-hepatectomy PVE. Future research should validate the causal role of MCART1 and ASB3 using genetic or pharmacological approaches and explore the clinical application of NR in liver surgery and metabolic liver disease.
